# Dramatic response to alectinib in a patient of ALK-rearranged lung cancer with poor performance status

**DOI:** 10.1186/s13104-016-1983-9

**Published:** 2016-03-17

**Authors:** Hisashi Tanaka, Kageaki Taima, Takeshi Morimoto, Kunihiko Nakamura, Yoshihito Tanaka, Masamichi Itoga, Shingo Takanashi, Ken Okumura

**Affiliations:** Department of Cardiology, Respiratory Medicine and Nephrology, Hirosaki University Graduate School of Medicine, Zaifu-cho 5, Hirosaki, 036-8562 Japan; Department of Laboratory Medicine, Hirosaki University School of Medicine, Honcho 53, Hirosaki, Japan; Department of Health Admnistration Center, Hirosaki University, Bunkyo 1, Hirosaki, Japan

**Keywords:** Lung cancer, Alectinib, Poor performance status, ALK

## Abstract

**Background:**

Lung cancers with anaplastic lymphoma kinase rearrangements are highly sensitive to anaplastic lymphoma kinase tyrosine kinase inhibition, underscoring the notion that such cancers are addicted to anaplastic lymphoma kinase activity. Several anaplastic lymphoma kinase inhibitors have been identified and are being evaluated in clinical trials. However patients with poor performance status (3 or 4) were not involved in these clinical trials, it has been unclear to use anaplastic lymphoma kinase–tyrosine kinase inhibitors for these patients. Here, we report an anaplastic lymphoma kinase-positive non small cell lung cancer patient with performance status 4, who was successfully treated with alectinib.

**Case presentation:**

We report on a 52-year-old patient diagnosed as non small cell lung cancer harboring echinoderm microtubule-associated protein-like 4-anaplastic lymphoma kinase fusion gene. His performance status was 4 because of severe respiratory failure. We treated this patient with alectinib as the first line therapy. Dramatic response was obtained and his performance status improved from 4 to 1 without severe adverse events.

**Conclusion:**

Alectinib is a therapeutic option for the anaplastic lymphoma kinase positive patients with poor performance status.

## Background

Non small cell lung cancer (NSCLC) is the leading cause of cancer-related deaths worldwide. Platinum-based chemotherapy is the standard therapy for patients with metastatic or recurrent disease. However, platinum-based chemotherapy is not recommended for patients with poor performance status (PS) of 3 or 4. Recently oncogenic drivers have been identified to play essential roles in the tumorigenesis, survival and proliferation in lung cancer. The National Comprehensive Cancer Network recommends epidermal growth factor receptor (EGFR) tyrosine kinase inhibitor (TKI) as a salvage treatment option for NSCLC patients with poor PS. While there has been a study looking at crizotinib versus chemotherapy in anaplastic lymphoma kinase (ALK) positive NSCLC [1], there is no prospective clinical evidence that indicates the efficacy of ALK TKIs for ALK positive patients with poor PS.

## Case report

**Fig. 1:**
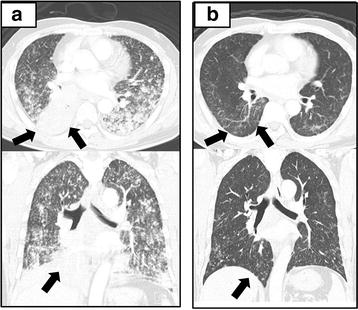
Computed tomography scan of the chest before the initiation of alectinib showing atelectasis in the *right lower* lung and multiple lung metastases (**a**). Computed tomography scan of the chest 3 weeks after the initiation of alectinib showing improvement of the atelectasis in the *right lower* lung and reduction of multiple lung metastases (**b**)

A 52-year-old man who was a smoker presented with severe respiratory failure. There was no special family history. A chest computed tomography (CT) showed a tumor in the right lower lung with atelectasis and multiple lung metastases in bilateral lung fields (Fig. 1a). Bone scintigraphy showed a hot spot on his lumber vertebrate, and thus clinical stage was IV (cT4N3M1b). As for his breathing state, SpO_2_ was around 90 % under the conditions of an oxygen flow rate 10 L/min with reservoir bag mask. His performance status (PS) was 4. Best supportive care seemed to be a proper choice. His medical history was unremarkable, and he had taken no medications. He had smoked 34 pack-years. The serum carcinoembryonic antigen (CEA) level was 8.6 ng/mL (normal range, 0–5.0 ng/mL). We conducted bronchoscopy by using high-flow nasal cannulae to make a pathological diagnosis and to assess molecular status. The diagnosis was adenocarcinoma, and moreover molecular analysis revealed that ALK protein was positive by immunohistochemical staining and *ALK* rearrangement was positive by reverse transcription polymerase chain reaction (RT-PCR). Fluorescence in situ hybridization (FISH) test was indeterminate.

**Fig. 2:**
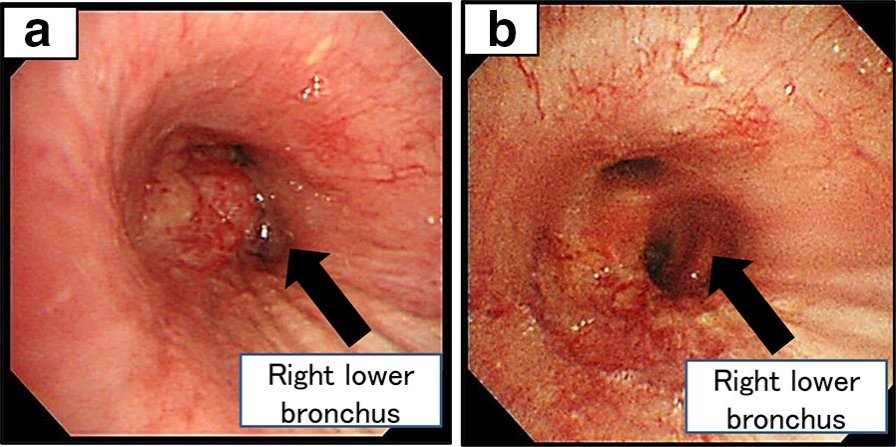
Bronchoscopy showing tumor obstruction in a visible range from the truncus intermedius to *right lower* lobe bronchus (**a**). Bronchoscopy taken 2 weeks after the initiation of alectinib showing dramatic shrinkage of *right lower* bronchus (**b**)

Therefore, we treated the patient with alectinib at 300 mg twice daily. In 3 weeks, chest CT showed dramatic shrinkage of the primary tumor in the right lower lobe and bilateral multiple lung metastases (Fig. 1b). Bronchoscopic study also showed dramatic improvement in right lower bronchus (Fig. 2a, b). The only adverse event was grade 1 elevation of alanine aminotransferase (ALT). The ALT level was 19 U/L before treatment, the maximum ALT level was 59 U/L during treatment. This adverse event was manageable. His PS improved from 4 to 1, the Karnofsy Performance Scale (KPS) improved from 10 to 90 %, and he discharged in 4 weeks his PS improved from 4 to 1 and he discharged in 4 weeks.

## Discussion

The echinoderm microtubule-associated protein-like 4 (*EML4*)-*ALK*, a fusion gene first described in 2007, possesses oncogenic activity that can be blocked by ALK inhibitor [2], and Rikova et al. [3] described two ALK fusion partners. Alectinib is a novel, highly selective oral ALK inhibitor [4, 5]. Seto et al. reported the efficacy and safety of alectinib in ALK inhibitor-naive patients with *ALK*-rearranged lung cancer in a phase 1–2 study. The overall response rate in the phase 2 part of the study was 93.5 %. The mean duration of treatment was 14.8 months, and at 1 year of follow-up, the median PFS had not been reached. The adverse events of alectinib were less than crizotinib [6]. There were no treatment-related grade 4 adverse events or deaths. Therefore, we chose alectinib for our patient. At present, crizotinib and alectinib are available as ALK inhibitors in Japan. The phase 3 studies (JapicCTI-132316, NCT02075840) comparing alectinib with crizotinib in the patients with chemo-naive, advanced *ALK*-rearranged lung cancer are ongoing. The superiority between the two drugs is not clear until the results of these trials come out. It should be pointed out that these clinical trials do not involve the patients with poor PS such as PS 3 or 4. In crizotinib, there are a few reports indicate the efficacy of crizotinib therapy for the patients with poor PS. Tamai et al. [7] reported a patient with ALK positive lung adenocarcinoma who was administered crizotinib via nasogastric and percutaneous endoscopic gastrostomy tubes. Ahn et al. [8] reported in the case of *EGFR*-mutated patients with poor PS, gefitinib is recommended as a first-line treatment. Inoue et al. [9] reported that the efficacy and feasibility of gefitinib for patients with poor PS and *EGFR* mutations. The objective tumor response rate was 66 % and toxicities were comparable to that observed in patients with PS 0–2. In the patients of an oncogene addicted lung cancer with poor PS, cytotoxic chemotherapy is not applicable, however, molecular target therapy might be feasible. Now, we can use two ALK-TKIs (crizotinib and alectinib) in Japan however, these ALK-TKIs have not been recommended in Japanese guideline because of no clinical evidence for such poor PS. To the best of our knowledge, this is the first case report of dramatic response to alectinib in an *ALK*-rearranged lung adenocarcinoma patient with PS 4.

## Conclusions

This case suggests alectinib could be a therapeutic option for such patients. Obviously, further study is necessary (UMIN000015094).

## Consent

Written informed consent was obtained from the patient for publication of this case report and accompanying images.

## Abbreviations

CT: computed tomography
PS: performance status
ALK: anaplastic lymphoma kinase
RT-PCR: reverse transcription polymerase chain reaction
FISH: fluorescence in situ hybridization
ALT: alanine aminotransferase
EGFR: epidermal growth factor receptor
